# Correspondence: Reply to ‘DNA shape is insufficient to explain binding'

**DOI:** 10.1038/ncomms15644

**Published:** 2017-06-05

**Authors:** Sivakanthan Kasinathan, Gabriel E. Zentner, Beibei Xin, Remo Rohs, Steven Henikoff

**Affiliations:** 1 Basic Sciences Division, Fred Hutchinson Cancer Research Center, Seattle, Washington 98109, USA; 2 Medical Scientist Training Program, University of Washington School of Medicine, Seattle, Washington 98195, USA; 3 Molecular and Cellular Biology Graduate Program, University of Washington, Seattle, Washington 98195, USA; 4 Department of Biology, Indiana University, Bloomington, Indiana 47405, USA; 5 Molecular and Computational Biology Program, University of Southern California, Los Angeles, California 90089, USA; 6 Howard Hughes Medical Institute, Fred Hutchinson Cancer Research Center, Seattle, Washington 98109, USA

*Nature Communications* 8:15644 doi: 10.1038/ncomms15644 (2017); Published 5 Jun 2017

Transcription factors (TFs) are DNA-binding proteins that regulate gene expression. Sequence-specific TFs recognize DNA via specific amino acid-base hydrogen bonds and contacts that read local DNA shape^1^. Studying base and shape readout modes of TFs *in vivo* has been challenging due to technical issues associated with current approaches for mapping TF-binding sites (TFBSs). We recently introduced Chromatin Endogenous Cleavage with sequencing (ChEC-seq), an *in vivo* mapping method based on fusing Micrococcal Nuclease (MNase) to a TF (ref. 2). Upon addition of calcium to permeabilized cells, tethered MNase cuts DNA adjacent to the bound TF and the released fragments are sequenced to provide a high-resolution genome-wide TFBS map. We used ChEC-seq to map the budding yeast TFs Abf1, Reb1 and Rap1 and obtained data similar to high-resolution ChIP-seq without the need for cross-linking, chromatin solubilization or antibodies.

When cells were collected <1 min after calcium addition, most TFBSs contained a TF-specific sequence motif (‘fast' sites). We also reported ‘slow' sites with low motif scores that appeared after ∼10 min. We found that DNA shape features of high-scoring (mostly fast) and low-scoring (mostly slow) TFBSs corresponded closely, but differed from randomly chosen sites not overlapping high- or low-scoring sites. In our study, DNA shape features of fast and slow sites were centred on the best match to the TF consensus motif; however, randomly chosen genomic intervals were not similarly centred on the best motif match. Rossi, Lai and Pugh now find that when random sites are motif-centred, the shape features correspond closely to slow site features^3^, which might suggest that DNA shape is insufficient to explain binding site selection by the TFs Abf1, Reb1 and Rap1. However, given that sequence and shape features covary^4^, it is problematic to rely on motif-dependent analyses to draw conclusions about whether a TF recognizes DNA shape^5^.

To address this problem, we aligned DNA shape feature vectors for unique fast and slow ChEC-seq sites for each TF using a procedure that relied only on shape data and was not directly informed by sequence alignment. Given the possibility for overlap between nearby TFBSs, we identified unique sites that do not intersect with any other ChEC-seq sites within intervals ranging from 100 to 500 bp surrounding ChEC-seq peak maxima, with larger windows associated with increasing stringency. For Abf1 and Reb1, we found that average fast and slow site shape features were well correlated at a range of interval widths (*P*<<0.001; Fig. 1a–c). We also searched sites using a ‘shape profile' defined using the average fast site features and found that score distributions for fast and slow sites only slightly differed (*P*>0.03), but were very different from random and free MNase sites (*P*<<10^−10^) for Abf1 (Fig. 1b) and Reb1 (not shown). The major shape feature proximal to Abf1 motifs is a deformation to the helix indicative of motif-proximal poly(dA:dT) tracts (Fig. 1a), a sequence feature we observed at slow sites in our original study^2^. Consistent with the recognition of a preferred shape signature by Abf1 and Reb1 at fast and slow sites, random sites and free MNase sites were not well correlated with fast and slow sites (Fig. 1a–c). We do not observe shape features enriched for poly(dA:dT) tracts at free MNase sites (Fig. 1a,b), suggesting that the detection of this shared shape feature at fast and slow ChEC-seq sites is not simply due to the higher prevalence of these features within nucleosome-depleted regions. Shape features at Rap1 fast and slow sites were not well correlated (*P<*0.1; Fig. 1c,d). The robustness of the correlation between average fast and slow shape features for Abf1 and Reb1 across a range of interval widths (Fig. 1d) suggests that sampling of similar shapes by TFs may explain binding events, even within promoters where fast and slow sites co-occur. From these motif-independent analyses, we conclude that fast and slow binding sites for Abf1 and Reb1 have similar shape features.

We next queried a TF-gene regulatory association database^6^, and asked whether TF-slow site associations had been previously observed in mapping or gene expression studies orthogonal to ChEC-seq. Consistent with our previous demonstration that slow sites were recovered as sites without the canonical motif in other studies^2^, the proportion of fast and slow sites documented or proposed to regulate proximal genes in previous studies (Fig. 1e) was similar across a range of interval widths. This suggests that slow sites with shape features similar to fast sites are likely true binding sites and not simply experimental noise due to cleavage proximal to fast sites.

What accounts for the differential sensitivity of these TFs to DNA shape? All three TFs are essential and have roles in maintaining nucleosome organization^7^,^8^; however, Rap1 is unique in that it also functions in chromatin silencing at the mating type locus and telomeres^9^. Promoter architecture in *Saccharomyces cerevisiae* may provide a basis for this functional specialization^4^. We observed marked deviations in DNA shape in the average aligned fast and slow site profiles for Abf1 (Fig. 1a) and Reb1, but not Rap1 (not shown) consistent with the presence of poly(dA:dT) tracts, which are known to exclude nucleosomes and play a role in establishing canonical chromatin architecture^4^,^10^. Abf1 and Reb1 have been proposed to be dependent on poly(dA:dT) tracts for their localization and function^11^,^12^,^13^. It has been suggested that poly(dA:dT) tracts may participate in regulating ribosomal protein gene promoters, which are also bound by Rap1 (ref. 14); however, our inability to detect significant DNA shape contributions to Rap1 binding may be due to the comparatively small number of sites tested. We speculate that promoters with poly(dA:dT) tracts not only exclude nucleosomes, but also have shape features that help recruit TFs that actively maintain nucleosome depletion^15^. Indeed, binding site-proximal poly(dA:dT) tracts have been proposed to enhance binding^16^, potentially by increasing accessibility of the adjacent major groove^17^. Thus, TF functional diversity and architecture of yeast promoters may explain the varying sensitivities of TFs to DNA shape. In this context, we anticipate that ChEC-seq will be a useful tool for generating high-resolution maps of protein-DNA interactions, with the potential to provide insights into the *in vivo* role of DNA shape in TFBS recognition.

## Methods

We defined unique sites such that the intersection of intervals of 100–500 bp widths centred on unique Abf1, Reb1, Rap1 and Free MNase ChEC-seq peak maxima was disjoint. As a null set, we generated 1,500 random intervals from the sacCer3 genome assembly that did not overlap with ChEC-derived peaks. Shape features in 201-bp windows centred on peak maxima were determined as described^4^ using the DNAshapeR package^18^. At each interval width for a given TF, sites that did not have overlapping shape alignment windows were selected for alignment. Motif-independent alignment involved comparing each site against every other site within a given class and determining the shift that maximized the cosine similarity. Within a class, all sites were aligned to an internal centroid, defined as the site with the smallest sum of squared cosine similarities versus all other sites. Sites were then shifted relative to the centroid and class-specific average features were computed. Pearson's *r* was used to quantify the similarity of average shape features between classes (reported *P* values are two-tailed) without shifting the average features relative to each other. Given the strong A/T MNase cleavage preference (not shown) in the 5-bp window centred on peak maxima, we excluded these positions from the alignment. Further, because shape readout likely occurs near the TFBS, the largest shift considered was 25 bp and alignment was limited to the 90-bp interval centred at the peak maximum. Parameters used for all site classes including the random and free MNase sites were identical. Shape profiles for Abf1 and Reb1 were defined as the regions in the average fast shape features with the largest information gain relative to shuffled sequences. Score distributions were generated by scoring the aligned fast, slow, free MNase and random sites in the same 90-bp interval used for shape alignment using correlation distance to the shape profile; Mann–Whitney U-tests were performed for pairwise comparisons of the resulting distributions. To determine whether putative TFBSs regulate nearby genes, we assigned them to their closest (≤1 kb) genes and queried YEASTRACT^6^. Source code for these analyses is publicly available (https://github.com/sivakasinathan/shape_align).

## Additional information

**How to cite this article:** Kasinathan, S. *et al*. Correspondence: Reply to ‘DNA shape is insufficient to explain binding'. *Nat. Commun.* **8,** 15644 doi: 10.1038/ncomms15644 (2017).

**Publisher's note**: Springer Nature remains neutral with regard to jurisdictional claims in published maps and institutional affiliations.

## Figures and Tables

**Figure 1:**
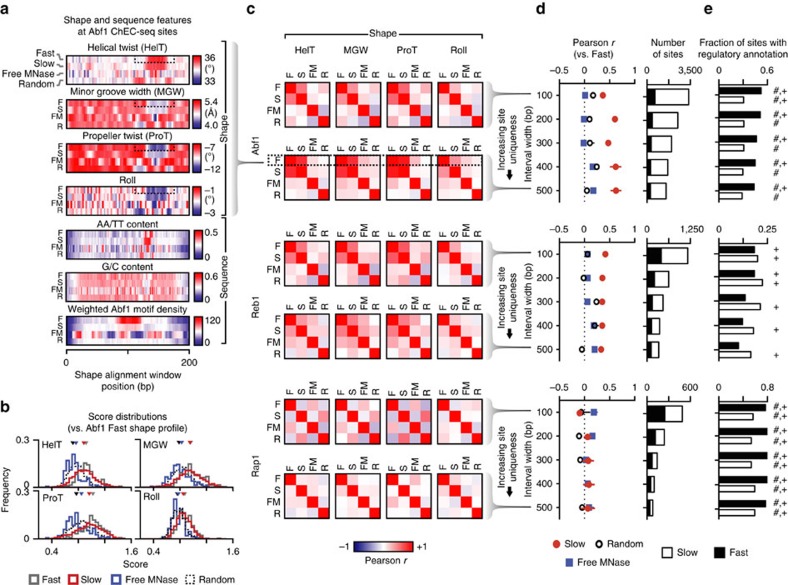
Slow ChEC-seq sites have characteristic shapes separate from TF-binding motifs. (**a**) Average shape and sequence features for unique fast (F) and slow (S) Abf1 sites 500 bp from other ChEC sites compared to free MNase (FM) and random (R) control sites aligned using shape features. Motif density was computed by weighting occurrences based on motif score. (**b**) Distributions of scores from searching shape vectors using the Abf1 fast site shape profile for fast and slow Abf1 sites (500 bp from other ChEC sites) and free MNase and random control sites; triangles represent the median score for each distribution. The shape profile used for searching is indicated in the dotted boxes in **a**. (**c**) Pearson correlations of average DNA helix twist (HelT), minor groove width (MGW), propeller twist (ProT), and roll features for unique fast and slow Abf1, Reb1 and Rap1 sites 100 bp (top) or 500 bp (bottom) from other ChEC sites compared to control sites aligned using shape features. (**d**) Pearson correlations compared to fast sites of aligned average DNA shape features at a variety of interval widths and the number of sites at each interval width. Error bars represent mean±s.e.m. (**e**) Proportion of unique sites at a range of overlap interval widths with known or proposed regulatory associations. *P* values<0.1 under Fisher's exact test versus FM and R control sites are indicated with ‘#' and ‘+', respectively.

## References

[b1] Rohs R. . Origins of specificity in protein-DNA recognition. Annu. Rev. Biochem. 79, 233–269 (2010). 20334529 10.1146/annurev-biochem-060408-091030 PMC3285485

[b2] Zentner G. E., Kasinathan S., Xin B., Rohs R. & Henikoff S. ChEC-seq kinetics discriminates transcription factor binding sites by DNA sequence and shape *in vivo*. Nat. Commun. 6, 8733 (2015). 26490019 10.1038/ncomms9733 PMC4618392

[b3] Rossi M. J., Lai W. K. M. & Pugh B. F. DNA shape is insufficient to explain binding. Nat. Commun. 8, 15643 (2017). 28580956 10.1038/ncomms15643 PMC5465349

[b4] Zhou T. . DNAshape: a method for the high-throughput prediction of DNA structural features on a genomic scale. Nucleic Acids Res. 41, W56–W62 (2013). 23703209 10.1093/nar/gkt437 PMC3692085

[b5] Krietenstein N. . Genomic nucleosome organization reconstituted with pure proteins. Cell 167, 709–721. e712 (2016). 27768892 10.1016/j.cell.2016.09.045 PMC5240917

[b6] Teixeira M. C. . The YEASTRACT database: an upgraded information system for the analysis of gene and genomic transcription regulation in Saccharomyces cerevisiae. Nucleic Acids Res. 42, D161–D166 (2014). 24170807 10.1093/nar/gkt1015 PMC3965121

[b7] Hartley P. D. & Madhani H. D. Mechanisms that specify promoter nucleosome location and identity. Cell 137, 445–458 (2009). 19410542 10.1016/j.cell.2009.02.043 PMC2677553

[b8] Ganapathi M. . Extensive role of the general regulatory factors, Abf1 and Rap1, in determining genome-wide chromatin structure in budding yeast. Nucleic Acids Res. 39, 2032–2044 (2011). 21081559 10.1093/nar/gkq1161 PMC3064788

[b9] Shore D. RAP1: a protean regulator in yeast. Trends Genet. 10, 408–412 (1994). 7809947 10.1016/0168-9525(94)90058-2

[b10] Struhl K. & Segal E. Determinants of nucleosome positioning. Nat. Struct. Mol. Biol. 20, 267–273 (2013). 23463311 10.1038/nsmb.2506 PMC3740156

[b11] Wu R. & Li H. Positioned and G/C-capped poly(dA:dT) tracts associate with the centers of nucleosome-free regions in yeast promoters. Genome Res. 20, 473–484 (2010). 20133331 10.1101/gr.103226.109 PMC2847750

[b12] Moreira J. M., Remacle J. E., Kielland-Brandt M. C. & Holmberg S. Datin, a yeast poly(dA:dT)-binding protein, behaves as an activator of the wild-type ILV1 promoter and interacts synergistically with Reb1p. Mol. Gen. Genet. 258, 95–103 (1998). 9613577 10.1007/s004380050711

[b13] Goncalves P. M. . Transcription activation of yeast ribosomal protein genes requires additional elements apart from binding sites for Abf1p or Rap1p. Nucleic Acids Res. 23, 1475–1480 (1995). 7784199 10.1093/nar/23.9.1475 PMC306885

[b14] Reja R., Vinayachandran V., Ghosh S. & Pugh B. F. Molecular mechanisms of ribosomal protein gene coregulation. Genes Dev. 29, 1942–1954 (2015). 26385964 10.1101/gad.268896.115 PMC4579351

[b15] Iyer V. & Struhl K. Poly(dA:dT), a ubiquitous promoter element that stimulates transcription via its intrinsic DNA structure. EMBO J. 14, 2570–2579 (1995). 7781610 10.1002/j.1460-2075.1995.tb07255.x PMC398371

[b16] Afek A., Sela I., Musa-Lempel N. & Lukatsky D. B. Nonspecific transcription-factor-DNA binding influences nucleosome occupancy in yeast. Biophys. J. 101, 2465–2475 (2011). 22098745 10.1016/j.bpj.2011.10.012 PMC3218343

[b17] Levo M. . Unraveling determinants of transcription factor binding outside the core binding site. Genome Res. 25, 1018–1029 (2015). 25762553 10.1101/gr.185033.114 PMC4484385

[b18] Chiu T.-P. . DNAshapeR: an R/Bioconductor package for DNA shape prediction and feature encoding. Bioinformatics 32, 1211–1213 (2016). 26668005 10.1093/bioinformatics/btv735 PMC4824130

